# Increasing incidence of central nervous system (CNS) tumors (2000–2012): findings from a population based registry in Gironde (France)

**DOI:** 10.1186/s12885-018-4545-9

**Published:** 2018-06-14

**Authors:** Camille Pouchieu, Anne Gruber, Emilie Berteaud, Patrice Ménégon, Pascal Monteil, Aymeri Huchet, Jean-Rodolphe Vignes, Anne Vital, Hugues Loiseau, Isabelle Baldi

**Affiliations:** 1 Equipe EPICENE, Centre INSERM U1219-Bordeaux Population Health Center, Université de Bordeaux, Bordeaux, France; 2 CHU de Bordeaux, Service de médecine du travail, Bordeaux, France; 3 CHU de Bordeaux, Service de neuro-imagerie diagnostique et thérapeutique, Bordeaux, France; 4 CHU de Bordeaux, Service de neurochirurgie, Bordeaux, France; 5 CHU de Bordeaux, Service de radiothérapie, Bordeaux, France; 6 CHU de Bordeaux, Laboratoire de neuropathologie, Bordeaux, France; 7 CHU de Bordeaux, Service de neurochirurgie B, Bordeaux, France

**Keywords:** Central nervous system neoplasms, Epidemiology, Incidence, Cancer registry, Trends, Meningiomas

## Abstract

**Background:**

Although some countries have observed a stabilization in the incidence of CNS, an increasing incidence has been reported from multiple studies. Recent observations point out to the heterogeneity of incidence trends according to histological subtypes, gender and age-groups. Using a high-quality regional CNS tumor registry, this article describes the trends of CNS tumor incidence for main histological subtypes, including benign and malignant tumors, in the French department of Gironde from 2000 to 2012.

**Methods:**

Crude and age-standardized incidence rates were calculated globally, by histological subtypes, malignant status, gender and age groups. For trends, annual percent changes (APC) were obtained from a piecewise log-linear model.

**Results:**

A total of 3515 CNS tumors was registered during the period. The incidence of overall CNS tumors was 19/100000 person-years (8.3/100000 for neuroepithelial tumors and 7.3/100000 for meningeal tumors). An increased incidence of overall CNS tumors was observed from 2000 to 2012 (APC = + 2.7%; 95%-confidence interval (CI): 1.8–3.7). This trend was mainly explained by an increase in the incidence of meningiomas over the period (APC = + 5.4%, 95%-CI: 3.8–7.0). The increased incidence rate of CNS tumors was more pronounced in female and in older patients even though the incidence rate increased in all age groups.

**Conclusions:**

Part of the temporal variation may be attributed to improvement in registration, diagnosis and clinical practices but also to changes in potential risk factors. Thus, etiological studies on CNS tumors are needed to clarify this rising trend.

**Electronic supplementary material:**

The online version of this article (10.1186/s12885-018-4545-9) contains supplementary material, which is available to authorized users.

## Background

Primary CNS tumors are a complex heterogeneous group of benign and malignant tumors, with more than 100 histologic subtypes of tumors from the brain to the spinal cord [1]. Compared to other sites of cancer, CNS tumors are rare in adults but they represent the second cause of cancer mortality in individuals aged < 19 years [2]. Estimating the burden of CNS tumors in the population requires considering heterogeneity in the trends according to histology, gender and age. From the 1970s to early 1990s, trends - generally described overall or only for gliomas - consistently showed an increase in the incidence rate in developed countries [3–9], especially in the elderly [10–12] and children [13–17]. Although the reason for this increase was debated, one explanation was that the ability to diagnose CNS tumors improved significantly after the introduction of computerized tomography scanning in the 1970s and magnetic resonance imaging in the early 1980s [7, 18]. A better registration of non-malignant CNS tumors and an improvement in clinical practice could have also contributed to this trend [19, 20]. Since the end of the 1990s, some registries reported continuous increase [4, 9, 21] but others tended to show that the incidence rate may be levelling off [14, 22] and may even decline [3, 5, 23–25]. Differences in the observations could also be explained by heterogeneity in the types of tumors or characteristics of the populations under study [26]. Indeed, recent data confirmed contrasted patterns of incidence trends for specific types of tumors and in specific age groups. On the 2000–2010 period in the US, the Central Brain Tumor Registry (CBTRUS) observed an increase in nonmalignant CNS tumors [27] since the Benign Brain Tumor Cancer Registries Amendment Act that made their collection mandatory [28]. The CBTRUS also observed an increase in malignant CNS tumors in children and adolescents, whereas no significant increase was seen in the whole population [27]. In the specific CNS tumor registry, operated in a French department (Gironde) in 1999, an increase in meningioma incidence rate on the 2000–2007 period was seen (Annual Percent Change (APC) + 5.4%), and also in neuro-epithelial tumors from 2003 (APC: + 7.5%) [21]. In the Nordic countries (Denmark, Finland, Norway, and Sweden), the incidence rate of glioma increased among those aged 60–79 years from 1974 to 2003, and after early 1990’s the incidence rate of meningioma increased among female, driven by the 60–79 year age group [22]. However, the incidence rate of childhood CNS tumors remained stable during 1985–2006 [29]. The UK cancer registries observed an increase in the incidence rate of CNS tumors from 1979 to 1992, mainly seen in the young (0–24 years) and the elderly (65–84 years), with different patterns of trends for specific types of tumors: meningiomas increased in those 25–84 years of age and pilocytic astrocytomas increased in the 0–24 years age group [10].

The aim of the present study was to provide a descriptive trend analysis of CNS tumor incidence rate from the Gironde CNS tumor registry, over the period 2000–2012 according to sex, age-group and main histologic subtypes.

## Methods

### Data collection

The French network of cancer registries has been previously described [21]. A cancer registry specifically collecting data about primary tumors of the CNS is based in Gironde, a department located in southwestern France. With an area of 9975 km^2^, it’s the largest French department. The population was estimated in 2012 to be 1,483,712 inhabitants (including 257,155 children and 92,327 adolescents). The sex ratio M/F was 0.92 and about 78% of the population lives in urban area (with a ratio urban/rural = 3.56). All neurosurgical activity was based in Bordeaux where the registry is located and no adjacent department had a neurosurgical institution. Expertise in pathology was mainly located in the University Teaching Hospital, which was the regional center of reference for public and private laboratories. Twenty-nine CT and 30 MRI machines are now available in the Gironde area, installed in the 1980s in the largest health centers. Patients from Gironde with suspected CNS tumors are unlikely to be referred outside the area, as the regional neurosciences center is based in Bordeaux.

From May 1999, all patients who lived in the department of Gironde in whom any new primary tumor of the CNS was diagnosed, whether symptomatic or asymptomatic, benign or malignant were prospectively registered. Spinal tumors were included. We excluded pituitary tumors, tumors associated with AIDS, recurrence of tumors, and metastatic tumors. No case was obtained from autopsy or from a death certificate only. Collection of the data is exhaustive because of the collaboration of a work group comprising practitioners (neurosurgeons, neuropathologists, medical oncologists, radiotherapists, neurologists, etc) involved in the diagnosis and therapeutic management of patients. To identify eligible cases and minimize the number of cases that may have been missed, multiple overlapping sources were used: (1) clinical reports obtained through registration forms filled in by practitioners, (2) extractions from the French National Hospital Database (PMSI) for relevant discharge data, (3) neuropatholology reports, (4) requests to the French Health Insurance Organization (Affections de Longue Durée) for free health treatment for CNS tumors, (5) death certificates, and (6) other cancer registries (The French National Registry of Childhood Solid Tumours and the Gironde Registry of Hematopoietic Malignancies).

Medical data were individually reviewed to ensure that the diagnoses were eligible, to check for the diagnosis date (from April 1999), and to fill in exclusion criteria. All duplicate cases were thoroughly searched and excluded. To ensure completeness, a periodic review of archives and pathology records was performed in the relevant departments and laboratories. Diagnosis was based on clinical and radiological data with or without histological confirmation. Whenever a surgical specimen was available for neuropathological analysis, the slides were systematically reviewed by a pathologist not involved in the initial diagnosis. When biopsy or surgical resection of the tumor was not possible, a monthly assessment was made by experienced neuroradiologists, neurosurgeons and radiotherapists to ascertain the diagnosis.

The following parameters were systematically recorded: date of birth, sex, postal code of usual residence, date of diagnosis, topography, and tumor histological subtype and grade. The usual residence corresponded to the address of cases at time of initial diagnosis of the tumor.

### Classification

Tumors were classified by grouping five-digit histology codes from the *International Classification of Diseases for Oncology, third edition*, into broad histology subgroups based on the recommendations from the 2000 Consensus Conference on Brain Tumor Definition for Registration [30].

### Statistical analyses

Overall, age- (five-year age groups) and sex-specific crude incidence rates (IR) were calculated per 100,000 inhabitants and per year. Population estimates by gender and calendar year were supplied by the Institut National de la Statistique et des Etudes Economiques (INSEE) (http://www.insee.fr/fr/bases-de-donnees/default.asp?page=recensement.htm). The study population was divided into children, adolescents and young adults (0–24 years), middle aged adults (25–49 years and 50–64 years separately), and elderly persons (65–80 years and ≥ 80 years separately). Rates were presented according to the rural/urban status assigned to the place of usual residence at time of initial diagnosis, obtained from the INSEE urban zoning classification (https://www.insee.fr/fr/information/2115011). Incidence rates were standardized on the 2000 US Standard Population [31], the Segi World Standard Population [32] and the Scandinavian “European” Standard Population [33] using the direct method of analysis to allow comparisons with other cancer registries. The annual age-standardized incidence rates (per 100,000) were calculated globally and separately for male and female.

For trends, a piecewise log linear model with constant variance and uncorrelated errors was run, using Joinpoint software (version 3.4.2) available on the Surveillance, Epidemiology and End Results (SEER)*Stat pages on the US National Cancer Institute website (https://surveillance.cancer.gov/joinpoint). The method implemented in the software allows choosing the number and the locations of joinpoints and testing whether an apparent change in trend is statistically significant. The test of significance uses a MonteCarlo permutation method. We considered two jointpoints because the number of data points was 13. The date of onset was aggregated into calendar years. We characterized trends in age and sex-standardized incidence rates using the French National census 2000–2012 by estimating APCs with 95% CIs. *P* < 0.05 was considered statistically significant. All tests were two-sided. Here we present data collected from January 2000 to December 2012.

## Results

### General characteristics

A total number of 3515 new primary CNS tumors was registered from 2000 to 2012, among the 1,483,712 inhabitants of Gironde, corresponding to a crude incidence rate of 19/100000 person-years, which was unchanged when standardizing on the French population (National census). To enable international comparisons, age-standardized incidence rates were calculated using the European Standard Population (Scandinavian, 1960) [33], the 2000 US Standard Population [31] and the World Standard Population (Segi’s, 1960) [32]. The age-standardized incidence rates of overall CNS tumors were 17/100000 (Europe), 17/100000 (US), and 13/100000 (World). Overall, 2706 tumors (77%) were histologically confirmed (from 63% for meningeal tumors to 89% for neuroepithelial tumors). Regarding the topography, most of the tumors were supratentorial (38.5%), 35.2% were meningeal, 14.9% were intracranial and intraspinal, 5.5% were unspecified, 4.2% were infratentorial and 1.7% were ventricular (data not tabulated).

### Incidence by sex, histological subtypes and malignancy status

The crude and age-standardized incidence rates of CNS tumors are presented in Table 1 by sex, histological subtypes and malignancy status. The crude incidence of CNS tumors was higher in female (21/100000) than in male (18/100000). Neuroepithelial tumors were the most frequent histological subtype of CNS tumors in this registry (*n* = 1496, 42.6%), followed by meningeal tumors (37.6%), tumors of the cranial and spinal nerves (12.0%), other tumors (4.6%) and lymphomas (3.3%). Histological subtypes are presented in more detail in Table 2. The most frequent subtypes of neuroepithelial tumors were glioblastomas (26.9% of CNS tumors) and mixed gliomas (2.8%). The histological subtypes of CNS tumors differed by sex: 55.2% of CNS tumors in male were neuroepithelial tumors whereas 49.0% of CNS tumors in female were meningeal tumors. The distribution and standardized incidence rates by age, sex and histological subtype are presented in Additional file 1: Table S1. In this CNS tumor registry, malignant tumors (44%) were less represented than non-malignant tumors (56%) and this differed by sex and histological subtypes. Malignant tumors were more frequent in male (56%) than in female (35%). Neuroepithelial tumors, cranial and spinal nerves tumors and lymphomas were mainly malignant tumors (91%, 99% and 100% respectively) and 98% of meningeal tumors were non-malignant tumors. Other tumors that combined different and rare histological subtypes included 73% of non-malignant tumors (data not shown).

**Table 1:** Crude and age-standardized incidence rate of primary CNS tumors according to sex, histological subtype and malignancy status^a^

	*n*	%	Crude IR/100,000	Standardized IR/100000		
				Europe	US	World
Men	1526		18	16	16	13
Malignancy status						
Malignant (/3)	860	56	9.9	9.1	9.2	7.3
Non-malignant (/1, /0)	666	44	7.7	7.2	7.2	5.9
Histological subtype						
Neuroepithelial tumors	843	55.2	9.7	9.1	9.1	7.3
Meningeal tumors	346	22.7	4.0	3.5	3.7	2.5
Cranial and spinal nerve tumors	208	13.6	2.4	2.3	2.2	1.8
Lymphomas	57	3.7	0.66	0.58	0.58	0.38
Other tumors	72	4.7	0.83	0.77	0.82	0.72
Female	1989		21	17	17	14
Malignancy status						
Malignant (/3)	688	35	7.3	5.9	5.9	4.6
Non-malignant (/1, /0)	1301	65	14	12	12	9.1
Histological subtype						
Neuroepithelial tumors	653	32.8	6.9	5.8	5.8	4.9
Meningeal tumors	975	49.0	10.4	8.4	8.4	6.5
Cranial and spinal nerve tumors	214	10.8	2.3	2.1	2.0	1.7
Lymphomas	58	2.9	0.62	0.45	0.47	0.35
Other tumors	89	4.5	0.94	0.72	0.77	0.68
All	3515		19	17	17	13
Malignancy status						
Malignant (/3)	1548	44	8.6	7.4	7.4	5.9
Non-malignant (/1, /0)	1967	56	11	9.5	9.6	7.6
Histological subtype						
Neuroepithelial tumors	1496	42.5	8.3	7.4	7.3	6.1
Meningeal tumors	1321	37.6	7.3	6.1	6.2	4.5
Cranial and spinal nerve tumors	422	12.0	2.3	2.2	2.1	1.8
Lymphomas	115	3.3	0.64	0.51	0.52	0.37
Other tumors	161	4.6	0.89	0.75	0.80	0.70

^a^Pituitary tumors, tumors associated with AIDS, recurrence of tumors, and metastatic tumors were not included in the registry

**Table 2:** Frequency and age and sex-standardized incidence rate of CNS tumors (reference: French National Census 2000–2012) according to histological subtype and sex^a^

Histology	ICDO-code	Male			Female			Total		
		n	%	IR	n	%	IR	n	%	IR
Neuroepithelial tumors										
Diffuse astrocytoma	9420/3	26	1.7	0.30	16	0.8	0.17	42	1.2	0.23
Anaplastic astrocytoma	9401/3, 9411/3	28	1.8	0.33	28	1.4	0.30	56	1.6	0.31
Glioblastoma	9440/3, 9441/3, 9442/3	528	34.6	6.1	417	21.0	4.2	945	26.9	5.3
Pilocytic astrocytoma	9421/1	17	1.1	0.20	17	0.9	0.19	34	1.0	0.19
Unique astrocytoma variants^b^	9383/1, 9384/1, 9424/3	12	0.8	0.14	8	0.4	0.09	20	0.6	0.11
Oligodendroglioma	9450/3	14	0.9	0.16	14	0.7	0.14	28	0.8	0.15
Anaplastic oligodendroglioma	9451/3	4	0.3	0.05	9	0.5	0.09	13	0.4	0.07
Ependymoma/anaplastic ependymoma	9391/3, 9392/3	37	2.4	0.43	19	1.0	0.20	56	1.6	0.31
Ependymoma variants (myxopapillary ependymoma)	9394/1	8	0.5	0.09	3	0.2	0.03	11	0.3	0.06
Mixed glioma^b^	9382/3	51	3.3	0.59	46	2.3	0.48	97	2.8	0.53
Astrocytoma, NOS	9400/3	20	1.3	0.23	11	0.6	0.12	31	0.9	0.17
Glioma malignant, NOS	9380/3	27	1.8	0.31	18	0.9	0.19	45	1.3	0.25
Choroid plexus	9390/0, 9390/1	6	0.4	0.07	4	0.2	0.04	10	0.3	0.06
Neuroepithelial	9381/3, 9430/3	4	0.3	0.05	7	0.4	0.08	11	0.3	0.06
Benign and malignant neuronal/glial, neuronal and mixed	8680/1, 9412/1, 9413/0, 9490/0,9492/0, 9500/3, 9505/1, 9505/3, 9506/1	32	2.1	0.37	25	1.3	0.26	57	1.6	0.31
Pineal parenchymal	9361/1, 9362/3	2	0.1	0.02	1	0.1	0.01	3	0.1	0.02
Embryonal/primitive/medulloblastoma	9470/3, 9471/3, 9473/3, 9508/3	27	1.8	0.32	10	0.5	0.11	37	1.1	0.21
Tumors of cranial and spinal nerves	9540/0, 9540/3, 9550/0, 9560/0	208	13.6	2.4	214	10.8	2.2	422	12.0	2.3
Meningeal Tumors										
Meningioma	9530/0, 9530/1, 9530/3, 9531/0, 9532/0, 9533/0, 9534/0, 9537/0, 9538/1, 9538/3, 9539/1	300	19.7	3.5	931	46.8	9.1	1231	35.0	6.8
Other mesenchymal, benign and malignant	8815/0, 8830/3, 8850/0, 8861/0, 8890/3, 9150/1, 9150/3, 9180/0, 9180/3	19	1.2	0.22	21	1.1	0.21	40	1.1	0.22
Hemangioblastoma	9161/1	27	1.8	0.31	23	1.2	0.23	50	1.4	0.27
Lymphomas	9590/3, 9591/3, 9680/3, 9731/3	57	3.7	0.66	58	2.9	0.58	115	3.3	0.64
Germ cell tumors and cysts^c^	9064/3, 9080/0, 9080/1, 9084/0, 9100/3	13	0.9	0.15	6	0.3	0.07	19	0.5	0.11
Tumors of the sellar region (craniopharyngiomas)	9350/1, 9351/1	21	1.4	0.24	19	1.0	0.20	40	1.1	0.22
Chordoma/chondrosarcoma	9320/0, 9220/3, 9370/3	3	0.2	0.04	10	0.5	0.11	13	0.4	0.07
Unclassified tumors	8000/1, 8000/3	35	2.3	0.40	54	2.7	0.46	89	2.5	0.49
Total		1526	100	18	1989	100	21	3515	100	19

*Abbreviations* : *IR* incidence rate, *NOS* not otherwise specified

^a^Pituitary tumors, tumors associated with AIDS, recurrence of tumors, and metastatic tumors were not included in the registry.

^b^Unique astrocytoma variants correspond to subependymoma (*n* = 12) subependymal giant cell astrocytoma (*n* = 7) and pleomorphic xanthoastrocytoma (*n* = 1).

^c^Mixed gliomas correspond to anaplastic oligoastrocytoma (*n* = 43) and oligoastrocytoma (*n* = 54); Germ cell tumors and cysts correspond to germinoma (*n* = 7), mature teratoma (*n* = 2), teratoma (*n* = 1), dermoïd cyst (*n* = 8), and choriocarcinoma (*n* = 1).

### Incidence rates by age and sex

**Fig. 1:**
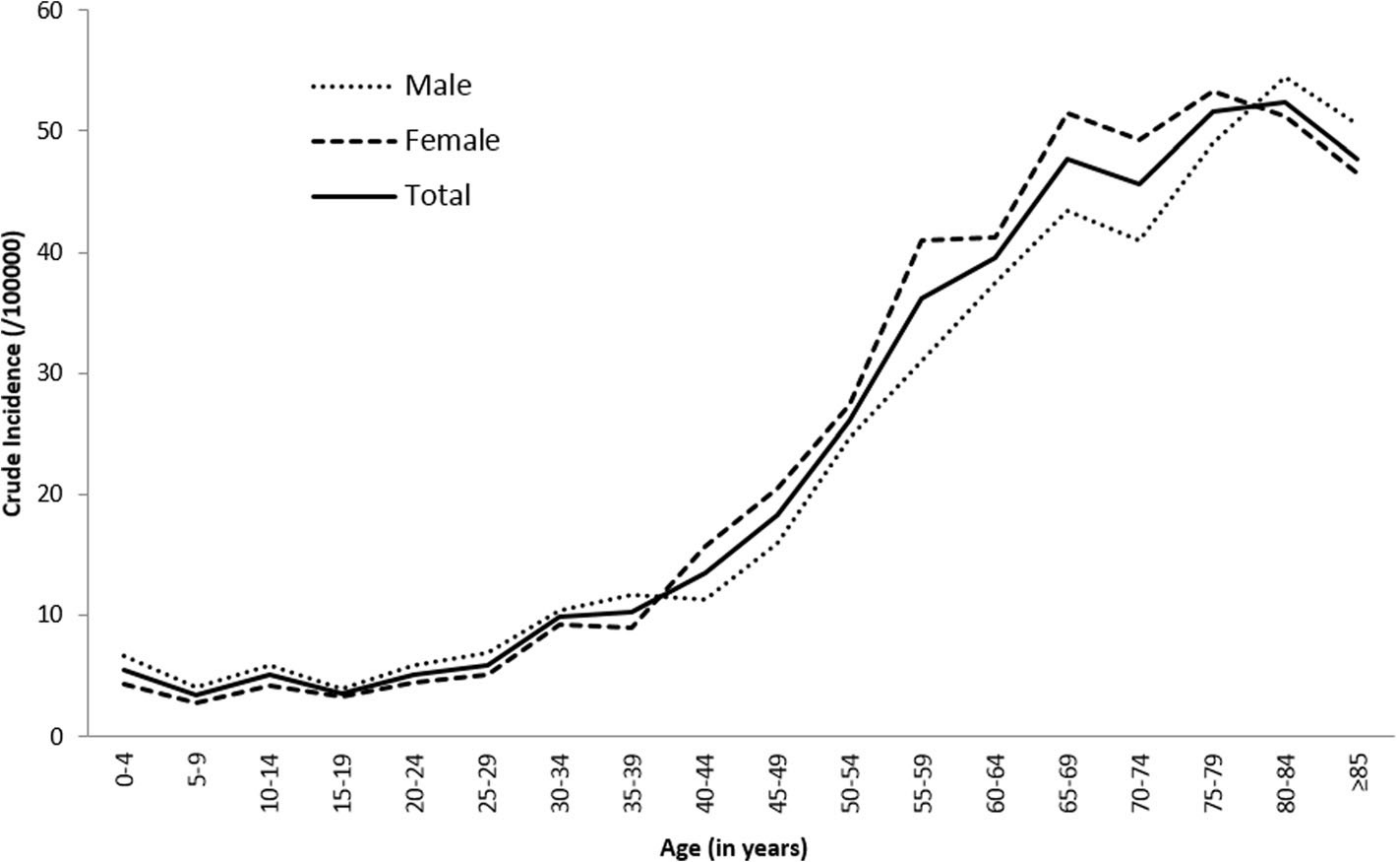
Crude incidence rates of central nervous system tumors by age and sex, Gironde CNS tumor registry, 2000–2012

Crude incidence rates of CNS tumors are presented by age and sex in Fig. 1. The incidence rate was 5.5/100000 in children less than 5 years, and ranged from to 3.4 to 5.0/100000 person-years in children aged 5–19 years. From 20 to 34 years of age, the incidence rate slightly increased from 5.1 to 9.8/100000. The incidence rate exceeded 10/100000 after 40 years, 25/100000 after 50, 35/100000 after 55, 45/100000 after 65, and finally culminated around 50/100000 after 70. Before 40 years of age, the incidence rate was higher in male than in female, then the opposite was observed until 80 years.

### Histological subtypes by age and sex

**Fig. 2:**
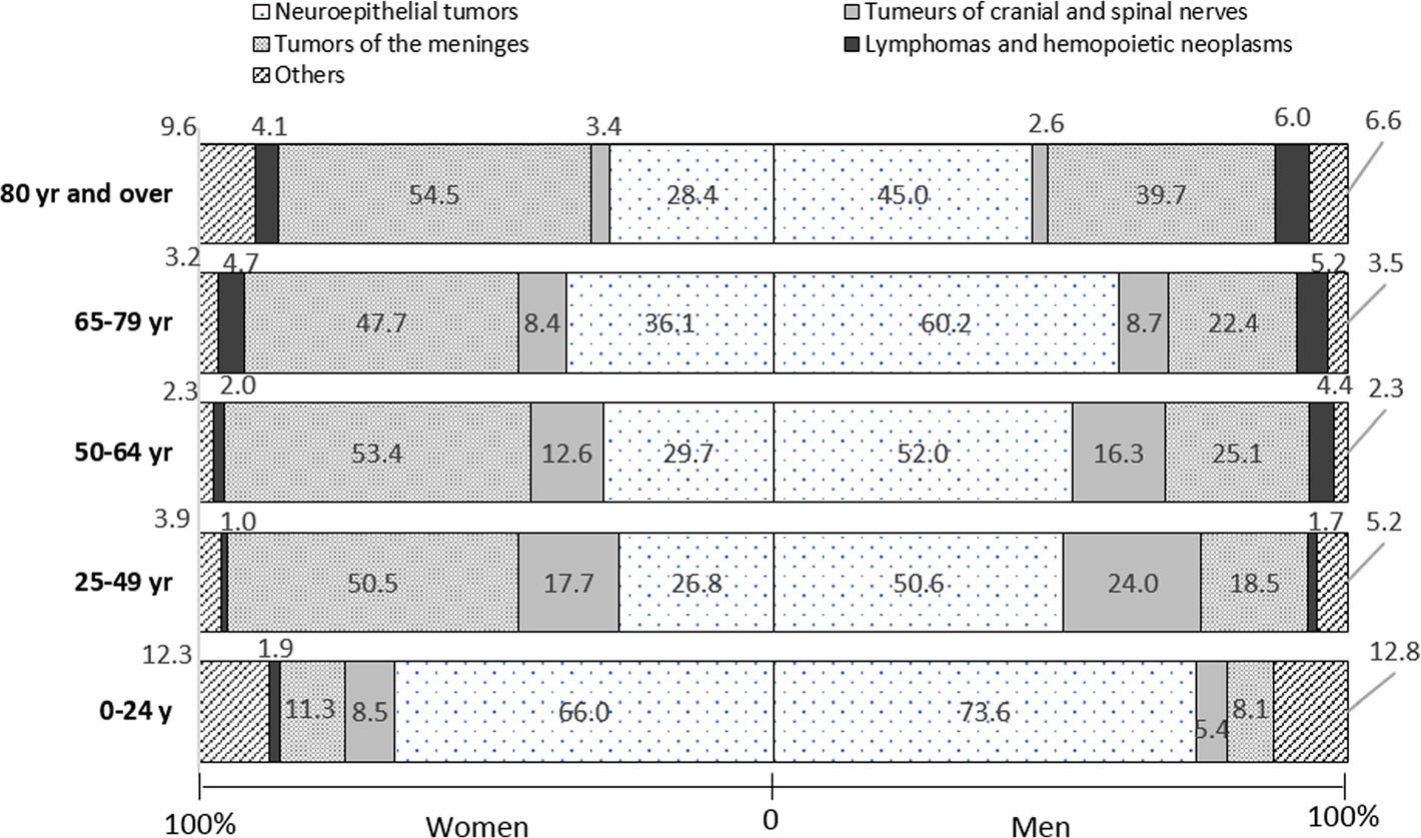
Distribution of main histological subtypes of central nervous system tumors by sex and age, Gironde CNS tumor registry, 2000–2012

The distribution of histological subtypes varied considerably according to age groups and sex (Fig. 2, Additional file 1: Table S1). In children, adolescents and young adults (0–24 years), neuroepithelial tumors were the tumors the most represented (IR = 3.2/100000) with a higher rate in boys than in girls (3.9 vs 2.5/100000), the incidence rate of meningeal tumors and tumors of the cranial and spinal nerves were 0.41/100000 and 0.30/100000, respectively, both with higher rates in girls than in boys. In this age group, the incidence rate of other tumors such as craniopharyngiomas, germ cell tumors and unclassified tumors was 0.57/100000 and the incidence rate of lymphomas was 0.04/100000 (0.07 in girls, no tumor registered in boys). In adults between 25 and 49 years, the incidence rate of neuroepithelial tumors was 4.4/100000 (5.7 in men, 3.2 in female) and that of meningeal tumors was 4.1/100000 (2.1 in men, 6.1 in female). The incidence rate of cranial and spinal nerve tumors was higher in male than in female (2.7/100000 vs 2.1). The incidence rates of all other types were less than 1/100000. In 50–64 years, the incidence rate of neuroepithelial tumors was similar to that of meningeal tumors (13.2/ 100,000 and 13.5/100000), the incidence rate of tumors of the cranial and spinal nerves were higher in male (5.0/100000) and female (4.5/100000) than in the previous age group. In persons aged 65–79 years, the incidence rate of neuroepithelial tumors was 22/100000 (27 in male, 18 in female) and that of meningeal tumors was 18/100000 (9.9 in male, 24 in female). The incidence rate of lymphomas was higher than in other age groups (2.3/100000 in male, 2.4 in female). In subjects aged 80 years and older, meningeal tumors were the most frequent type of CNS tumors (incidence rate = 25/100000) (26 in female, 21 in male), followed by neuroepithelial tumors (17/100000) and other tumors (4.3/100000).

### Trends over the 13-year period

#### All CNS tumors

**Fig. 3:**
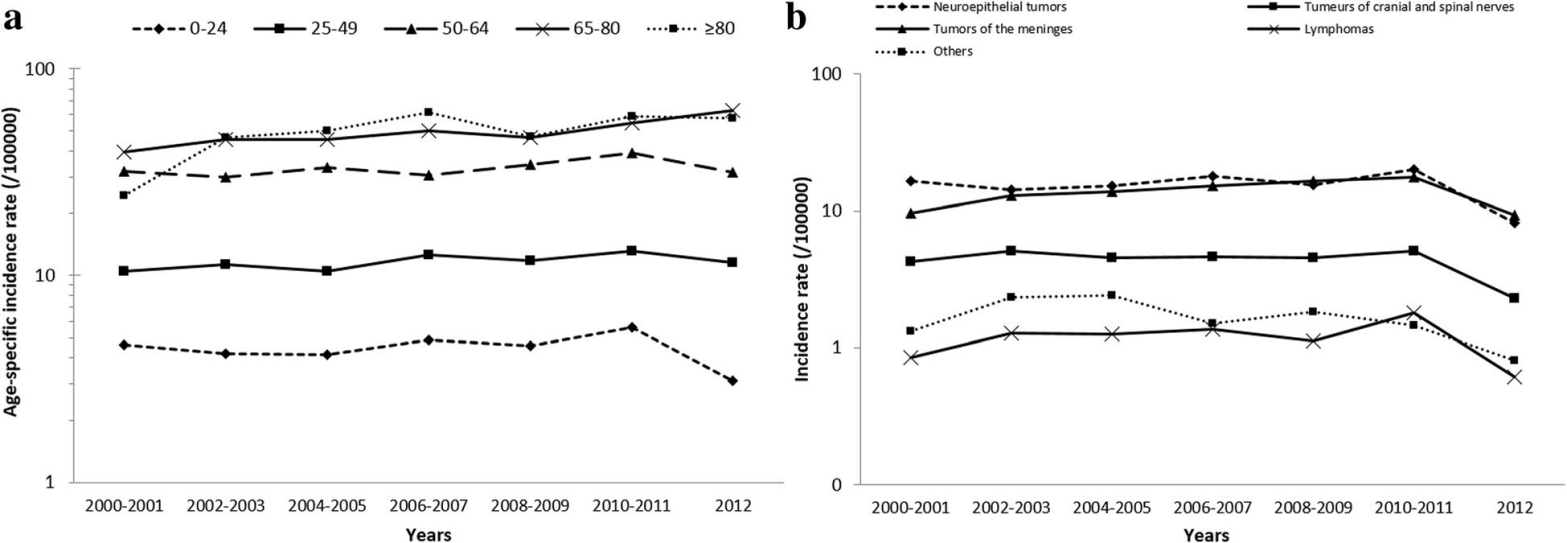
**a** Trends in age-specific incidence rate of central nervous system tumors, Gironde CNS tumor registry, 2000–2012. **b** Trends in crude incidence rate of central nervous system tumors by histological subtype, Gironde CNS tumor registry, 2000–2012

From 2000 to 2012, the APC for overall CNS tumors was + 2.7% and was statistically significant (*p* < 0.0001) (Table 3). The increase of the age-standardized incidence rates (using the French National Census as reference population) was higher in female (+ 3.6%, *p* < 0.0001) than in male (+ 1.8%, *p* = 0.006). The IRs in the age groups younger than 65 years were quite higher during the period. An increase in incidence rate was observed in all age groups (Fig. 3a), but significant only in the 65 to 79-year-old age group on the whole period (+ 3.0%, *p* = 0.002). The changes in the rates over the period did not appear to be related to the usual place of residence at the time of diagnosis, as the APC was similar in urban (+ 2.4%) and rural (+ 4.0%) settings, nor to the rate of histological confirmation, as the annual increase was observed in tumors both with (+ 2.1%, *p* < 0.0001) and without (+ 4.8%, *p* < 0.0001) histological confirmation.

**Table 3:** Annual percent change in age and sex-standardized incidence rates of primary CNS tumors (reference: French National Census 2000–2012) according to sex, age, histology, malignancy status and urban/rural place of residence, Gironde CNS registry, 2000–2012

	*n*	period	APC	95% CI, APC	Trend *P*-value
All CNS Tumors^a^	3515	2000–2012	+ 2.7	(+ 1.8; + 3.7)	< 0.0001
Sex					
Male	1526	2000–2012	+ 1.8	(+ 0.3; + 3.3)	0.006
Female	1989	2000–2012	+ 3.6	(+ 2.2; + 5.0)	< 0.0001
Age					
0–24 y	254	2000–2012	+ 0.6	(−2.5; + 3.8)	0.7
25–49 y	730	2000–2012	+ 1.2	(− 0.9; + 3.4)	0.2
50–64 y	1088	2000–2012	+ 1.2	(− 0.6; + 3.1)	0.2
65–79 y	1000	2000–2012	+ 3.0	(+ 0.8; + 5.2)	0.002
80 y and over^b^	443	2000–2002	+ 62	(+ 27; + 108)	< 0.0001
		2002–2012	+ 1.4	(− 2.4; + 5.4)	0.4
Place of residence					
Urban	2835	2000–2012	+ 2.4	(+ 1.6; + 3.2)	< 0.0001
Rural	680	2000–2012	+ 4.0	(+ 1.4; + 6.8)	0.0008
Histological confirmation					
Yes	2706	2000–2012	+ 2.1	(+ 1.1; + 3.2)	< 0.0001
No	809	2000–2012	+ 4.8	(+ 2.8; + 6.9)	< 0.0001
Malignant tumors(/3)	1548	2000–2012	+ 1.3	(− 0.20; + 2.8)	0.06
Sex					
Male	860	2000–2012	+ 0.99	(− 0.82; + 2.8)	0.2
Female	688	2000–2012	+ 1.7	(−0.42; + 3.9)	0.1
Age					
0–24 y	122	2000–2012	+ 0.90	(− 3.5; + 5.5)	0.6
25–49 y	268	2000–2012	− 1.2	(− 5.9; + 3.7)	0.6
50–64 y	457	2000–2012	+ 1.4	(− 1.3; + 4.2)	0.2
65–79 y	513	2000–2012	+ 1.3	(−1.4; + 4.0)	0.3
80 y and over	188	2000–2012	+ 2.0	(−4.0; + 8.4)	0.5
Non-malignant tumors (/1, /0)	1967	2000–2012	+ 4.0	(+ 2.7 − + 5.3)	< 0.0001
Sex					
Male	666	2000–2012	+ 3.1	(+ 0.35; + 5.8)	0.01
Female	1301	2000–2012	+ 4.6	(+ 2.6; + 6.7)	< 0.0001
Age					
0–24 y	132	2000–2012	+ 0.53	(−3.3; + 4.5)	0.8
25–49 y	462	2000–2012	+ 2.8	(+ 0.35; + 5.3)	0.01
50–64 y	631	2000–2012	+ 1.3	(−1.3; + 3.9)	0.3
65–79 y	487	2000–2012	+ 4.8	(+ 2.3; + 7.4)	< 0.0001
80 y and over^b^	255	2000–2002	+ 103	(+ 5.1; + 292)	0.01
		2002–2012	+ 4.5	(−0.70; + 10)	0.05
Neuroepithelial tumors	1496	2000–2012	+ 1.5	(−0.34; + 3.5)	0.1
Sex					
Male	843	2000–2012	+ 1.5	(−0.64; + 3.6)	0.1
Female	653	2000–2012	+ 1.6	(−0.54; + 3.9)	0.1
Age					
0–24 y	179	2000–2012	+ 1.3	(−3.8; + 6.6)	0.6
25–49 y	278	2000–2012	−1.1	(−5.9; + 4.0)	0.6
50–64 y	430	2000–2012	+ 1.6	(−0.95; + 4.2)	0.2
65–79 y	458	2000–2012	+ 1.5	(−1.5; + 4.6)	0.3
80 y and over	151	2000–2012	+ 3.7	(−1.9; + 9.7)	0.1
Meningeal tumors	1321	2000–2012	+ 5.4	(+ 3.8; + 7.0)	< 0.0001
Sex					
Male	346	2000–2012	+ 6.1	(+ 0.82; + 11)	0.01
Female	975	2000–2012	+ 5.6	(+ 3.4; + 7.8)	< 0.0001
Age					
0–24 y	24	2000–2012			
25–49 y	258	2000–2012	+ 4.2	(+ 0.75; + 7.8)	0.007
50–64 y	445	2000–2012	+ 1.5	(−1.1; + 4.2)	0.2
65–79 y	375	2000–2012	+ 5.8	(+ 2.5; + 9.2)	< 0.0001
80 y and over	219	2000–2012	+ 11	(+ 5.1; + 17)	< 0.0001
Other histological subtypes					
Cranial and spinal nerve tumors	422	2000–2012	+ 0.68	(−0.75; + 2.1)	0.3
Lymphomas	115	2000–2012	+ 3.6	(−3.5; + 11)	0.3
Other tumors^c^	161	2000–2012	− 1.2	(−6.6; + 4.5)	0.6

*CI* confidence interval, *APC* annual percent change

^a^Pituitary tumors, tumors associated with AIDS, recurrence of tumors, and metastatic tumors were not included in the registry

^b^The joinpoint analysis identified two trends for individuals 80 years and over: 2000–2002 and 2002–2012

^c^Other tumors included germ cell tumors, tumors of the sellar region and unclassified tumors

#### According to malignancy status and histological subtypes

No time trend was found for malignant CNS tumor but an increase in non-malignant tumors was observed (+ 4.0%), slightly higher in female (+ 4.6%, *p* < 0.0001) than in male (+ 3.1%, *p* = 0.01) and specifically in patients aged 65–79 years (+ 4.8, *p* < 0.0001).

A non-significant increase was found for neuroepithelial tumors (+ 1.5%) (Fig. 3), similar in both sexes (+ 1.5% in men, + 1.6% in female), observed in all age groups (except the 25–49 years), and slightly more pronounced in individuals 80 years and over (APC: + 3.7, *p* = 0.15). A more pronounced increase was observed for meningeal tumors, with an APC of + 5.4% for the whole period, which was statistically significant (*p* < 0.0001). This trend was slightly higher in male (+ 6.1%, *p* = 0.01) than in female (+ 5.6%, *p* < 0.0001), existed for all age groups except those 50–64 years old but tended to be higher in elderly: the APC was + 4.2% in the 25 to 49 year old group, + 5.8 in the 65 to 79 year age group and reached + 11% in the group 80 years and older (*p* = 0.007, *p* < 0.0001, *p* < 0.0001 respectively). No time trend was found for cranial and spinal nerve tumors and unstable results were obtained for lymphomas and other tumors owing to the limited number of cases over the 13 years period.

## Discussion

The Gironde CNS Tumor Registry that reports all histological subtypes, including benign and malignant tumors, provides reliable data on CNS tumor incidence rate in the southwestern area of France with the longest period of follow-up available for specific types of tumors such as meningioma. The incidence rate of overall CNS tumors was 19/100000 person-years on the period 2000–2012 and the age-standardized rates were 17, 17 and 13/100000 using the European Standard Population (Scandinavian 1960), the 2000 US Standard Population and the World Standard Population (Segi, 1960), respectively, which was higher than observed in other cancer registries [9, 23, 34–41]. The distribution of age groups was quite similar between the US and the European standard populations explaining similar rates. However, the lowest rate was observed standardizing on the World Standard Population since this population was younger than other standards (40% was aged < 20 years and 11% was aged ≥60 years in the World standard vs 29% and 16% in others standards). Large geographical and temporal variations have been observed in the incidence rate of CNS tumors ranging from 5.9 to 21/100000 according to cancer registries, due to the limitations and heterogeneity of registration procedures. However, few data has been published over a comparable period and mainly focused on malignant CNS tumors, which makes comparisons difficult. For malignant tumors, the CBTRUS found a 8.9/100000 incidence rate standardized on the 2000 US standard population [27] and the New Zealand Cancer Registry observed a 6.7/100000 incidence rate standardized on the WHO standard population [25], which were comparable to the rates we found in Gironde when focusing on malignant CNS tumors (7.4/100000 when standardized on the 2000 US standard population). The most comparable data derived from the Austrian Brain Tumor Registry, which found an age-standardized incidence rate (US) of 18/100000, corresponding to 16/100000 when excluding tumors of the sellar region [34]. Concerning meningioma, the incidence rate standardized on the 2000 US standard population was slightly lower to that found by the SEER Program (6.2 in Gironde vs 7.6/100000 in the US) [42].

The global characteristics of CNS tumors included in the Gironde Tumor Registry were consistent with most other studies [9, 26, 43, 44]. The incidence rate of overall CNS tumors increased dramatically with age, neuroepithelial tumors were the histological subtype the most represented in male whereas meningeal tumors were the most represented in female.

From 2000 to 2012, we observed an increase in the incidence rate of CNS tumors (APC = 2.7%), which was explained mainly by an increase in the incidence rate of meningioma over the period (APC = + 5.4%). The overall increase in the incidence rate of CNS tumors was more pronounced in female and in elderly persons. Other authors have found an overall increase in CNS tumors, especially in older ages even if they did not detect clear changes in specific subgroups [9, 10, 22, 39]. In the elderly, the overall increase may be attributed to the increase of meningioma incidence rate over the entire period (+ 11%, *p* < 0.0001). We can notice that this rising trend in meningioma is outstanding compared to other publications, which may be partly explained by the willingness of clinicians to pursue a diagnosis in older patients. This result is in line with the finding that the incidence rate of meningioma increased 3.9-fold from 1943 to 1997 in Denmark [45]. Arora observed that the incidence rate of meningioma significantly increased in England for the period 1979–2003 with the highest increases in those 65–84 years (+ 2.9%) [10]. Dolecek has also found a 3.8 increase in benign meningioma in elderly persons ≥75 years in the United States from 2004 to 2011 [42].

The temporal trend we observed may have several explanations: improvement in tumor registration, diagnosis and clinical practice although no significant change in the registration procedure or in access to imagery technologies was observed during this period. The indication of surgical procedures has changed over time. As more patients undergo surgery, the number of histological confirmation increases. However, this should have a minimal impact in our study as there was an increased incidence rate in tumors both with and without histological confirmation, which was twice as higher for tumors without histological confirmation (APC = + 2.1 and + 4.8, respectively). However, we cannot rule out that the use of three new sources of tumor registration from 2005 to 2007 may contribute to this rising trend although this is unlikely to explain the difference between sex and histological subtypes.

Even if the role of environmental risk factors (pesticides, electromagnetic fields) was not well-established, changes in potential risk factors may also be responsible for this temporal trend. With about 135,000 ha of vineyards and 28,000 individuals involved in wine-growing in the department, it cannot be excluded that these findings may be explained by environmental risk factors, notably by the potential role of pesticides that are largely used in vineyards. Indeed, a case-control study conducted in Gironde found that a high level of occupational exposure to pesticides might be associated with an excess risk of brain tumors, especially of gliomas [46]. The last 20 years, the prevalence of obesity in France have been increasing steadily, including in the South-West area [47]. Recently, a meta-analysis of 12 cohorts and case-control studies found that overweight and obesity have been associated with increased risk of meningiomas [48]. Thus, we cannot exclude that the body mass index may partly contribute to the increasing trend in meningioma incidence rate. In addition, mobile phones were introduced in the 1980’s and became widespread by the early 1990s. There is growing concern that the exposure to mobile phone use might increase the risk of CNS tumors. In 2013, the International Agency for Research on Cancer (IARC) classified radiofrequencies generated by mobile phones as possible human carcinogens on the basis of epidemiological data that showed an increased risk of gliomas [49]. The predominance of meningioma for female also suggests a potential role of hormonal factors. Indeed, higher incidence rate of meningioma has been observed in female of reproductive age, in tumor expression of hormone receptors, and changes in the size of meningioma during pregnancy, the menstrual cycle and menopause [50]. However, apart from ionizing radiation that has been classified as an agent with sufficient evidence of carcinogenicity in humans for cancers of the brain and CNS (Group 1) by the IARC [51], the role of these factors remain unresolved and further investigation is needed.

## Conclusions

Although the incidence rate of CNS tumors has tended to level off in some countries, we highlighted an overall increase in CNS tumor incidence rate in the French department of Gironde, during a recent 13 years period (2000–2012), especially for meningiomas. The present analysis also pointed at several patterns of incidence trends for CNS tumors, according to histological subtypes, sex and age groups, suggesting potential changes in lifestyle, environmental or occupational risk factors of CNS tumors. The increase in meningioma over the period was in line with recent findings from other studies. Consistently, the overall increase was more pronounced in female and in elderly persons but the reasons for the trends remain unclear. Thus, etiological studies which aims at elucidating the potential role of environmental and occupational exposures (including pesticides and electromagnetic fields) on brain tumors are needed to clarify this rising trend.

## Additional file

Additional file 1: **Table S1.** Distribution and standardized incidence rates (reference populations Europe, US, world) by age, sex and histological type, Gironde CNS registry, 2000–2012. (DOCX 18 kb)

## Abbreviations

APC: annual percent change
CBTRUS: Central Brain Tumor Registry of the United-States
CNS: central nervous system
IARC: International Agency for Research on Cancer
INSEE: Institut National de la Statistique et des Etudes Economiques
PMSI: Programme de médicalisation des systèmes d’information
SEER: Surveillance, Epidemiology and End Results
