# Supplementary material for: Increasing incidence of central nervous system (CNS) tumors (2000–2012): findings from a population based registry in Gironde (France)
Source: BMC Cancer. 2018 Jun 14;18:653. doi: 10.1186/s12885-018-4545-9 (PMC6001067; doi:10.1186/s12885-018-4545-9)
Supplement: Supplementary file 1 — Table S1. Distribution and standardized incidence rates (reference populations Europe, US, world) by age, sex and histological type, Gironde CNS registry, 2000–2012. (DOCX 18 kb) [file 12885_2018_4545_MOESM1_ESM.docx]

Additional Table 1. Distribution and standardized incidence rates (reference populations Europe, US, world) by age, sex and histological type, Gironde CNS registry, 2000-2012

|  |  |  | | **0-24 y** | | | |  | | **25-49 y** | | | |  | | **50-64 y** | | | |  | | **65-79 y** | | | |  | | **80 y and over** | | | |
| --- | --- | --- | --- | --- | --- | --- | --- | --- | --- | --- | --- | --- | --- | --- | --- | --- | --- | --- | --- | --- | --- | --- | --- | --- | --- | --- | --- | --- | --- | --- | --- |
|  |  | % |  | | Standardized IR/ | | | % |  | | Standardized IR/ | | | % |  | | Standardized IR / | | | % |  | | Standardized IR / | | | % |  | | Standardized IR / | | |
|  |  |  |  | | 100 000 | | |  |  | | 100 000 | | |  |  | | 100 000 | | |  |  | | 100 000 | | |  |  | | 100 000 | | |
|  |  |  | France | | Europe | US | World |  | France | | Europe | US | World |  | France | | Europe | US | World |  | France | | Europe | US | World |  | France | | Europe | US | World |
| Neuroepithelial tumours | **Men** | 42.9 | 3.9 | | 3.8 | 3.9 | 3.9 | 24.0 | 5.7 | | 5.7 | 5.7 | 5.6 | 22.9 | 16 | | 16 | 16 | 16 | 24.2 | 27 | | 27 | 27 | 26 | 15.3 | 24 | | 24 | 24 | 24 |
|  | **Women** | 27.6 | 2.5 | | 2.5 | 2.5 | 2.5 | 14.1 | 3.2 | | 3.2 | 3.2 | 3.1 | 16.6 | 11 | | 11 | 11 | 11 | 21.6 | 18 | | 18 | 18 | 18 | 18.7 | 14 | | 14 | 14 | 14 |
|  | **All** | 70.5 | 3.2 | | 3.2 | 3.2 | 3.2 | 38.1 | 4.4 | | 4.4 | 4.5 | 4.3 | 39.5 | 13 | | 13 | 13 | 13 | 45.8 | 22 | | 22 | 22 | 22 | 34.1 | 17 | | 17 | 17 | 18 |
| Cranial and spinal nerves tumours | **Men** | 3.1 | 0.28 | | 0.28 | 0.28 | 0.27 | 11.4 | 2.7 | | 2.7 | 2.7 | 2.6 | 7.2 | 5.0 | | 5.0 | 4.9 | 4.9 | 3.5 | 3.9 | | 4.0 | 3.9 | 4.0 | 0.9 | 1.4 | | 1.6 | 1.4 | 1.6 |
|  | **Women** | 3.5 | 0.30 | | 0.33 | 0.30 | 0.29 | 9.3 | 2.1 | | 2.1 | 2.1 | 2.0 | 7.1 | 4.5 | | 4.4 | 4.5 | 4.5 | 5.0 | 4.3 | | 4.6 | 4.6 | 4.7 | 2.3 | 1.7 | | 1.7 | 1.6 | 1.7 |
|  | **All** | 6.7 | 0.29 | | 0.31 | 0.29 | 0.28 | 20.7 | 2.4 | | 2.4 | 2.4 | 2.3 | 14.2 | 4.7 | | 4.7 | 4.7 | 4.7 | 8.5 | 4.1 | | 4.3 | 4.3 | 4.4 | 3.2 | 1.6 | | 1.7 | 1.5 | 1.6 |
| Meningial tumours | **Men** | 4.7 | 0.43 | | 0.42 | 0.43 | 0.43 | 8.8 | 2.1 | | 2.1 | 2.1 | 2.0 | 11.0 | 7.6 | | 7.9 | 7.7 | 7.8 | 9.0 | 9.9 | | 9.7 | 9.7 | 9.6 | 13.5 | 21 | | 21 | 21 | 21 |
|  | **Women** | 4.7 | 0.41 | | 0.44 | 0.41 | 0.41 | 26.6 | 6.1 | | 6.0 | 6.1 | 5.6 | 29.9 | 19 | | 19 | 19 | 19 | 28.5 | 24 | | 24 | 25 | 25 | 35.9 | 27 | | 26 | 26 | 26 |
|  | **All** | 9.4 | 0.42 | | 0.43 | 0.42 | 0.42 | 35.3 | 4.1 | | 4.1 | 4.1 | 3.8 | 40.9 | 13 | | 14 | 14 | 13 | 37.5 | 18 | | 18 | 18 | 18 | 49.4 | 25 | | 24 | 24 | 24 |
| Lymphomas | **Men** | 0 | 0 | | 0 | 0 | 0 | 0.8 | 0.20 | | 0.20 | 0.20 | 0.20 | 1.9 | 1.3 | | 1.3 | 1.3 | 1.3 | 2.1 | 2.3 | | 2.3 | 2.4 | 2.4 | 2.0 | 3.2 | | 3.3 | 3.3 | 3.3 |
|  | **Women** | 0.8 | 0.07 | | 0.06 | 0.07 | 0.07 | 0.5 | 0.13 | | 0.12 | 0.12 | 0.11 | 1.1 | 0.71 | | 0.72 | 0.70 | 0.70 | 2.8 | 2.4 | | 2.4 | 2.4 | 2.4 | 2.7 | 2.0 | | 2.1 | 2.0 | 2.1 |
|  | **All** | 0.8 | 0.04 | | 0.03 | 0.04 | 0.03 | 1.4 | 0.16 | | 0.16 | 0.16 | 0.15 | 3.0 | 1.01 | | 1.00 | 1.00 | 0.99 | 4.9 | 2.4 | | 2.4 | 2.4 | 2.4 | 4.7 | 2.4 | | 2.4 | 2.4 | 2.5 |
| Other tumors | **Men** | 7.5 | 0.67 | | 0.69 | 0.67 | 0.66 | 2.5 | 0.59 | | 0.59 | 0.59 | 0.59 | 1.0 | 0.69 | | 0.69 | 0.70 | 0.71 | 1.4 | 1.5 | | 1.5 | 1.5 | 1.5 | 2.3 | 3.4 | | 3.3 | 3.7 | 3.5 |
|  | **Women** | 5.1 | 0.48 | | 0.47 | 0.48 | 0.48 | 2.1 | 0.47 | | 0.47 | 0.47 | 0.50 | 1.3 | 0.85 | | 0.90 | 0.85 | 0.86 | 1.9 | 1.6 | | 1.6 | 1.6 | 1.6 | 6.3 | 4.7 | | 4.7 | 4.7 | 4.7 |
|  | **All** | 12.6 | 0.57 | | 0.58 | 0.58 | 0.57 | 4.5 | 0.53 | | 0.53 | 0.53 | 0.55 | 2.3 | 0.77 | | 0.80 | 0.78 | 0.79 | 3.3 | 1.6 | | 1.6 | 1.6 | 1.5 | 8.6 | 4.3 | | 4.3 | 4.3 | 4.3 |

IR = incidence rates; y = years
